# Genetically Induced Tumors in the Oncopig Model Invoke an Antitumor Immune Response Dominated by Cytotoxic CD8β^+^ T Cells and Differentiated γδ T Cells Alongside a Regulatory Response Mediated by FOXP3^+^ T Cells and Immunoregulatory Molecules

**DOI:** 10.3389/fimmu.2018.01301

**Published:** 2018-06-07

**Authors:** Nana H. Overgaard, Daniel R. Principe, Kyle M. Schachtschneider, Jeanne Toft Jakobsen, Laurie A. Rund, Paul J. Grippo, Lawrence B. Schook, Gregers Jungersen

**Affiliations:** ^1^Department of Micro- and Nanotechnology, Technical University of Denmark, Kongens Lyngby, Denmark; ^2^Department of Animal Sciences, University of Illinois at Urbana-Champaign, Champaign, IL, United States; ^3^Medical Scientist Training Program, University of Illinois College of Medicine, Chicago, IL, United States; ^4^Department of Radiology, University of Illinois, Chicago, IL, United States; ^5^Department of Biotechnology and Biomedicine, Technical University of Denmark, Kongens Lyngby, Denmark; ^6^Department of Medicine, University of Illinois at Urbana-Champaign, Chicago, IL, United States

**Keywords:** porcine cancer model, comparative oncology, translational immunology, antitumor immunity, T cells, immunotherapy

## Abstract

In recent years, immunotherapy has shown considerable promise in the management of several malignancies. However, the majority of preclinical studies have been conducted in rodents, the results of which often translate poorly to patients given the substantial differences between murine and human immunology. As the porcine immune system is far more analogous to that of humans, pigs may serve as a supplementary preclinical model for future testing of such therapies. We have generated the genetically modified Oncopig with inducible tumor formation resulting from concomitant *KRAS^G12D^* and *TP53^R167H^* mutations under control of an adenoviral vector Cre-recombinase (AdCre). The objective of this study was to characterize the tumor microenvironment in this novel animal model with respect to T-cell responses in particular and to elucidate the potential use of Oncopigs for future preclinical testing of cancer immunotherapies. In this study, we observed pronounced intratumoral T-cell infiltration with a strong CD8β^+^ predominance alongside a representation of highly differentiated γδ T cells. The infiltrating CD8β^+^ T cells displayed increased expression of the cytotoxic marker perforin when compared with the peripheral T-cell pool. Similarly, there was robust granzyme B staining localizing to the tumors; affirming the presence of cytotoxic immune cells within the tumor. In parallel with this antitumor immune response, the tumors displayed enrichment in FOXP3-expressing T cells and increased gene expression of indoleamine 2,3-dioxygenase 1 (*IDO1*), cytotoxic T-lymphocyte-associated protein 4 (*CTLA4*), and programmed death-ligand 1 (*PDL1*). Finally, we investigated the Oncopig immune system in mediating antitumor immunity. We observed pronounced killing of autologous tumor cells, which demonstrates the propensity of the Oncopig immune system to recognize and mount a cytotoxic response against tumor cells. Together, these findings suggest innate and adaptive recognition of the induced tumors with a concomitant *in vivo* suppression of T-cell effector functions. Combined, the data support that the Oncopig may serve as a valuable model for future preclinical testing of immunotherapies aimed at reactivating tumor-directed cytotoxicity *in vivo*.

## Introduction

For decades, preclinical studies pertaining to novel cancer therapies have relied on animal models of disease. Traditionally, rodents have been the gold standard for cancer research providing invaluable insights into the interplay between the immune system and tumor cells. However, despite these numerous advances, mice often failed to fully recapitulate human cancers, and many promising preclinical therapies were unsuccessful in the clinic (1, 2). Beyond differences in disease pathogenesis and progression between rodents and humans (3–5), the size constraints of rodents often do not support the investigation of new surgical interventions (4, 6). In light of the numerous obstacles presented by rodent models of disease, alternative model systems have been proposed, including zebrafish (7, 8), cats (9), dogs (9–14), and pigs (15–22). Due to homology in physiology, anatomy, size, genetics, metabolism, life span, and immunome between humans and pigs (15, 23–25), a porcine model may be extremely relevant for preclinical testing of cancer treatments. Furthermore, in contrast to murine cells, both porcine and human somatic cells demonstrate suppressed telomerase activity in most tissues that is reactivated during cancer development (26, 27). For this reason, induction of oncogenesis in humans and pigs generally requires a greater number of genetic defects than in mice (3, 6). To determine the relevance of the pig as a preclinical platform for immunotherapy, we employed the Oncopig model with inducible oncogenic RAS and dominant-negative P53 (28). Upon exposure to an adenoviral vector Cre-recombinase (AdCre), the infected cells of the transgenic Oncopig acquire two driver mutations: *KRAS^G12D^* and *TP53^R167H^*; two of the most common genetic abnormalities in human cancer (28, 29). The ability of tumor cells to avoid immune destruction has been included as a hallmark of tumorigenesis (30). Toward this end, immune checkpoint inhibitors have demonstrated tremendous promise in the clinic (31–33). However, when predicting patient responsiveness to such immunotherapies, the number and types of intratumoral immune cells are key factors (34–37). The Immunoscore suggests a new classification of cancer, where the tumor microenvironment plays an important role, and the relationship between intratumoral immune cells and patient prognosis is taken into account (38–40). This new approach currently serves as a prognostic tool for colorectal cancer; however, the universal applicability of the Immunoscore as a prognostic strategy in various cancer types remains to be fully validated (41). Given the importance of the intratumoral immune cells in both prognosis and response to therapy, we performed a characterization of the immunological landscape in Oncopig tumors to evaluate the applicability of the model for studying antitumor immune responses and for future testing of immunotherapies in a large and relevant *in vivo* system.

## Materials and Methods

### Pigs

The *KRAS^G12D^* and *TP53^R167H^* floxed Oncopigs (28) were neither sex- nor age-matched, and all animals were housed at the University of Illinois, Urbana-Champaign, United States. F1 animals (minipig carrying the transgene crossed with Yorkshire domestic pigs) heterozygous for the transgenes were used for experiments. A total of 27 animals were included. All animal experiments were carried out in accordance with both national and international guidelines. The University of Illinois Institutional Animal Care and Use Committee (IACUC; Protocol number 14126) approved all procedures.

### AdCre Injections for Tumor Induction

All animals were anesthetized using an intramuscular injection of Telazol^®^-Ketamine-Xylazine, 1 ml/50 lbs. The AdCre (Ad5CMVCre-eGFP, Gene Transfer Vector Core, University of Iowa, batch: Ad3500 or Ad3743, cat. no. VVC-U of Iowa-1174) was used for triggering tumors *in vivo*, and the preparation was previously described elsewhere (28, 42). Briefly, AdCre was diluted with minimal essential medium (Corning, cat. no. 50-011) containing 2 M calcium chloride resulting in a final concentration of calcium chloride of 0.01 M. Following dilution, the final concentration of AdCre ranged from 1 × 10^9^ to 2 × 10^9^ PFU/ml. The mixture was allowed to incubate at room temperature (RT) for 15 min before injection. For all subcutaneous injections (flank), a total volume of 1 ml AdCre was injected. For intramuscular injections (leg), animals received 0.5 or 1 ml. All AdCre injections were carried out using a 21 gauge needle and completed within 45 min from the time of incubation. Each animal received between one and six AdCre injections at the same time to induce one or multiple tumors. Animals were monitored every second day, and tumor measurements was carried out using a caliper. All animals were euthanized 7–21 days post AdCre injection; the exact time was depended on tumor size. For euthanasia, pigs were injected intracardially with 1 ml/5 kg body weight of Fatal-Plus^®^ Solution (Vortech Pharmaceuticals, cat. no. 9373).

### Immunohistochemistry

Tissues were fixed in 10% formalin and paraffin-embedded. Slides were sectioned at 4 µm interval and all subsequent steps were carried out at RT. Heat-induced epitope retrieval was carried out using a Menarini Access Retrieval Unit with a sodium citrate buffer (pH 6) for 1 min 40 s at 125°C, full pressure. The slides were then loaded onto a Dako Autostainer and rinsed with a Tris/Tween buffer (pH 7.5) before treatment with Dako Real TM Peroxidase blocking solution (Agilent Technologies, cat. no. S202386-2) for 5 min followed by buffer rinse (Tris/Tween, pH 7.5) for an additional 5 min. Slides were then treated with the primary antibody: Polyclonal Rabbit Anti-Human CD3 (Agilent Technologies, cat. no. A045201-2) diluted in Dako universal diluent (Agilent Technologies, cat. no. S080981-2) and stained for 30 min. Two rounds of 5 min buffer rinse (Tris/Tween, pH 7.5) were carried out before secondary staining with Dako EnVision + System-HRP Labeled Polymer Anti rabbit (Agilent Technologies, cat. no. K400211-2) for 30 min. The slides were then rinsed twice (Tris/Tween, pH 7.5) and treated with 3,3′-diaminobenzidine + substrate-chromogen system (Agilent Technologies, cat. no. K346889-2) for 10 min. Finally, the slides were washed thrice in H_2_O and counterstained with Gills Hematoxylin (Sigma-Aldrich, cat. no. GHS1128) for 27 s followed by additional wash in H_2_O.

### Immunofluorescence

Tissues were fixed in 10% formalin, embedded in paraffin, and sectioned at 4 µm intervals. For immunofluorescence, slides were heated in a pressure cooker using DAKO Target Retrieval Solution (Agilent Technologies, cat. no. S170084-2), blocked for 1 h at RT with Innovex Background Buster (Innovex, cat. no. NB306) with 5% Fc Receptor Block (Innovex, cat. no. NB309) and incubated with primary antibodies against CD3 (Santa Cruz Biotech, cat. no. sc-20047), CD8α (Santa Cruz Biotech, cat. no. sc-7188), or Granzyme B (abcam, cat. no. ab134933) at 1:100–200 overnight at 4°C. Slides were mounted in a DAPI containing medium (Santa Cruz) and visualized using either Alexa Fluor 488 (abcam, cat. no. ab150113) or Alexa Fluor 594 (abcam, cat. no. ab150080) conjugated secondary antibodies.

### Cell Isolation

Peripheral blood samples were collected from the jugular vein using BD sodium heparinized vacutainer tubes (BD Diagnostics, cat. no. 362753) and purified using SepMate tubes (StemCell Technologies, cat. no. 85450) according to the manufacturer’s protocol. Briefly, sodium heparinized blood was diluted 1:1 in phosphate buffered saline (PBS)/2% fetal bovine serum (FBS) (Thermo Fisher Scientific, cat. no. 10082147) before separation using Lymphoprep (StemCell Technologies, cat. no. 07851) with centrifugation settings at 12,00 × *g* for 20 min at 4°C. Cells were subsequently washed twice and counted using a hemocytometer. Viable cells were distinguished from dead cells using Trypan blue (Sigma-Aldrich, cat. no. T0887). To isolate cancer cells from *in vivo*-induced tumors; a 1 cm^3^ tumor biopsy was harvested and cut into small pieces before incubation in pre-heated RPMI-1640 containing 2% FBS, 3 mg/ml Collagenase D (Sigma-Aldrich, cat. no. COLLD-RO), 5 µg/ml DNase I (Sigma-Aldrich, cat. no. 11284932001), and 1 µg/ml Dispase II (Sigma-Aldrich, cat. no. 04942078001) for 90 min at 37°C. Samples were vortexed every 30 min to facilitate digestion. Cells were then passed twice through a 70 µm cell strainer to obtain a single-cell suspension. Processing was completed within 6 h for all cells. Cells were counted using the Nucleocounter NC-200 (Chemometec, Allerød, Denmark), and 10^7^ cells per vial of peripheral blood mononuclear cells (PBMCs) or tumor cells were cryopreserved for subsequent analysis. FBS/10% dimethyl sulfoxide (DMSO) was used as freezing medium, and every vial was placed in a Mr. Frosty freezing container at −80°C within 3 min of exposure to DMSO. The vials were transferred to liquid nitrogen 24 h later for long-term storage.

### Flow Cytometry

Antibodies were used at pre-determined optimal concentrations (Table S1 in Supplementary Material). Cryopreserved PBMCs and tumor cell suspensions were thawed in RPMI-1640/20% FBS and subsequently washed twice in PBS/0.5% FBS. The median viability post thawing was 91.7% as determined by the Nucleocounter NC-200, and ~4 × 10^6^ cells per sample were stained for flow cytometry. The samples were then surface stained for 30 min at 4°C with a combination of anti-CD3, anti-CD4, anti-CD8α, anti-CD8β antibodies, and a live/dead stain allowing viable cells to be distinguished from dead cells. For detection of γδ T cells, thawed cell suspensions were stained with a combination of anti-CD2, anti-TCR1 δ chain, anti-CD8α, and a live/dead stain. For detection of FOXP3, cells were fixed and permeabilized post surface staining using the Anti-Mouse/Rat Foxp3 Staining Set (Thermo Fisher Scientific, cat. no. 72-5775-40) according to the manufacturer’s protocol. Cells were then incubated with anti-FoxP3 antibody for 30 min at 4°C. In performing intracellular cytokine staining, samples were first cultured for 16 h at 37°C, 5% CO_2_ in RPMI-1640/10% FBS medium; serum was pretested in cell stimulation assays before use. As a positive control, 1 µg/ml phytohemagglutinin (Sigma-Aldrich, cat. no. L4144) was used for stimulation. To block cytokine secretion, cells were then cultured for an additional 6 h in the presence of 10 µg/ml Brefeldin A (Sigma-Aldrich, cat. no. B7651-5MG). Following surface stain with antibodies (Table S1 in Supplementary Material), cells were then fixed using the Fixation/Permeabilization Solution Kit (BD Biosciences, cat. no. 554714) according to the manufacturer’s protocol, stained with a mixture of anti-IFN-γ, anti-TNF-α, and anti-perforin antibodies for 30 min at 4°C. KRAS^G12D^ was detected by flow cytometry using the Fixation/Permeabilization Solution Kit directly with no pre-culturing in the presence of Brefeldin A. In all staining procedures, fluorescence-minus-one controls were included. Samples were acquired using an LSR II (BD Biosciences, Albertslund, Denmark) or an LSRFortessa (BD Bioscience, Albertslund, Denmark) flow cytometer, and the PMT voltages were adjusted based on a mixture of unstained cells resulting in a mean auto fluorescence intensity of ~10^2^ for all fluorochromes. Outputs were analyzed using either FCS Express version 6 (De Novo Software) or FlowJo Data Analysis Software version 10. The analysis was performed on viable, single cells (lymphocytes or tumor cells) (Figure S1A in Supplementary Material) with the following gating strategy being indicated in each figure legend. Examples of CD3, CD4, CD8β, CD8α, perforin, and FOXP3 staining are shown in Figure S1B in Supplementary Material. All samples (a minimum of 200,000 T cells) were recorded for analysis.

### *In Vitro* Cytotoxicity

Freshly isolated PBMCs and tumor cells were washed twice with PBS to remove any serum and counted using the hemocytometer and Trypan Blue. Effector cells (PBMCs) remained unlabeled. Control cells (30 × 10^6^ PBMCs) and target cells (30 × 10^6^ isolated tumor cells) were labeled with 10 µM eFluor450^®^ and 5 µM eFluor670^®^ Cell Proliferation Dye (eBioscience, cat. no. 65-0842-85 and 65-0840-85), respectively, according to the manufacturer’s protocol. Briefly, cells were labeled for 10 min at 37°C in the dark and labeling was stopped by adding four to five volumes of cold RPMI-1640/10% FBS. The cells were then incubated on ice for 5 min covered in the dark followed by three washing steps with RPMI-1640/10% FBS. For culturing, a titration of effector:target cell ratio was carried out as follows: 0:1, 0.5:1, 1:1, and 2:1; culturing conditions were 37°C, 5% CO_2_ in 24-well plates. Each well contained a total of 3 × 10^6^ cells. Samples were harvested at 10 min and 24 h post coculturing, fixed immediately with a 4% paraformaldehyde solution (Fisher Scientific, cat. no. 199431LT) to eliminate additional killing or cell turnover. Notably, culture wells containing effector:control cells and effector:target cells were mixed only at the time of harvesting; samples were then fixed to stop potential additional killing or cell turn over and acquired straight away on the flow cytometer. Samples were washed twice in PBS/0.5% FBS and acquired using an LSR II (BD Biosciences) flow cytometer, and data were analyzed using FCS Express version 6 (De Novo Software). PMT voltages were once again adjusted according to an unstained sample; the mean auto fluorescence value for each fluorochrome was adjusted to approximately 10^2^. For each sample, ~1.5 × 10^6^ cells were acquired for analysis. The percentage of specific killing was determined by comparing the percentage change ratio between control and target cell populations at baseline and 24 h post coculture. Individual animal values were normalized to background levels of killing/cell turnover from wells with no-effector cells added.

### RNA-Seq Analysis

Previously RNA-Seq datasets were produced for Oncopig primary hepatocyte cell lines (*n* = 3), transformed hepatocyte [hepatocellular carcinoma (HCC)] cell lines (*n* = 3), primary fibroblast cell lines (*n* = 8), and transformed fibroblast (soft-tissue sarcoma) cell lines (*n* = 4) and were downloaded from the ENA database^1^ under accession number PRJEB8646 (43, 44). In addition, previously produced Oncopig skeletal muscle (*n* = 3) and leiomyosarcoma tumor (*n* = 4) RNA-Seq datasets were downloaded from the ArrayExpress database^2^ under accession number E-MTAB-3382 (28). All datasets consisted of paired-end 100 bp reads produced on an Illumina HiSeq2000 (E-MTAB-3382) or Illumina HiSeq2500 (PRJEB8646). Sequencing depths for each sample are provided in Table S3 in Supplementary Material. Raw reads were trimmed, aligned to the swine reference genome (45), and assessed for differential gene expression as previously described in Ref. (28, 43, 44). Briefly, reads were trimmed sequentially for adapter contamination, A-tails, and minimum quality score (20) and length (20 bp) using Trim Galore v.0.3.3,^3^ setting the stringency option to 6. Trimmed reads were then aligned to the swine reference genome (Sscrofa10.2) using Tophat v.2.2.10 (46) with the -M, -G, fr-firststrand option, and setting the read-realign-edit-dist option to 0. Aligned bam files were assessed for differential gene expression using cufflinks v.2.2.1 (47). Transcripts were assembled using the fr-firststrand option and merged with the swine reference transcripts using Cuffmerge. Cuffdiff was used to assess differential expression for three comparisons (hepatocyte cell lines vs HCC cell lines, primary fibroblast cell lines vs soft-tissue sarcoma cell lines, and skeletal muscle vs leiomyosarcoma tumors) using the -u and fr-firststrand options. Genes were considered differentially expressed with a *q*-value < 0.05.

### Statistical Analysis

Despite low numbers of animals, the data were analyzed by parametric analyses as 80% of datasets showing a significant difference to baseline data passed the Shapiro–Wilk normality test. Results are shown as the mean ± SEM. Statistical comparisons of mean values were conducted using either paired or unpaired Student’s *t*-test depending on the experimental setup. All statistical analysis was carried out using GraphPad Prism version 7.00 for Windows (CA, USA). *P < 0.05 was considered significant. **P < 0.005 and ***P < 0.001 are indicated. To take the false discovery rate into account, *q*-values rather than *P*-values were used for RNA-Seq analysis (44, 48). A *q*-value < 0.05 was considered significant.

## Results

### *KRAS^G12D^*-Expressing Tumors Are Infiltrated by T Cells

**Figure 1:**
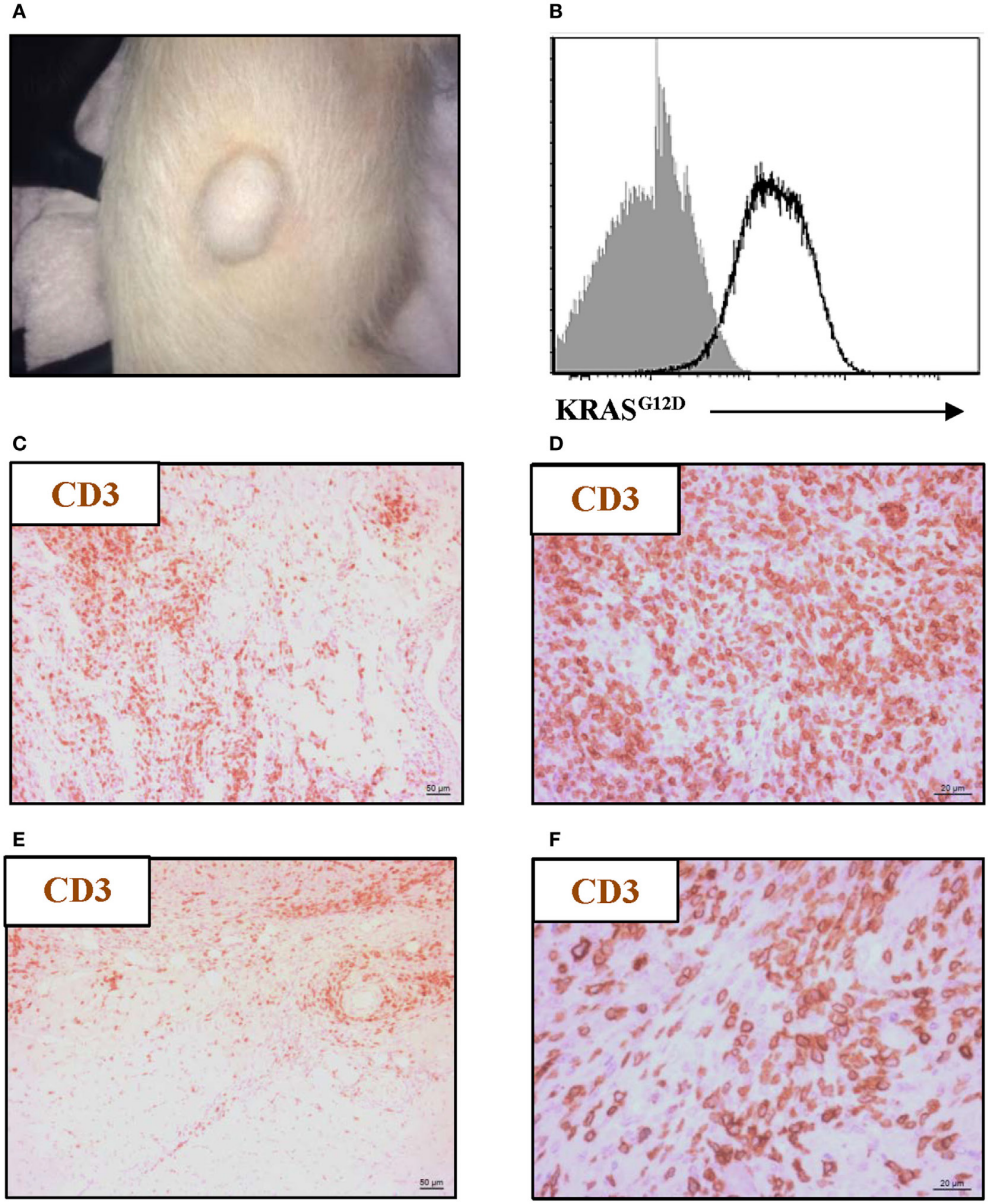
Oncopig tumors are infiltrated by T cells. The *KRAS^G12D^* and *TP53^R167H^* floxed Oncopigs were subcutaneously injected with AdCre to induce tumorigenesis. **(A)** Representative image of subcutaneous tumor formation in Oncopigs 7–21 days post subcutaneous injection into the flank of AdCre (*n* = 6), where *n* indicates the number of animals. **(B)** Representative intracellular flow cytometric plot of *KRAS^G12D^-*expression in isolated tumor cells (white) with fluorescence-minus-one control indicated (gray). Oncopigs were subcutaneously into the flank **(C,D)** or intramuscularly into the leg **(E,F)** injected with AdCre, and tumor sections were harvested 20 days post injection. Representative immunohistochemistry images with detection of CD3^+^ cells at 10× **(C,E)** and 40× **(D,F)** magnifications are shown (*n* = 3), where *n* indicates the number of animals.

To confirm tumorigenesis in this porcine model, Oncopigs were subcutaneously injected into the flank with AdCre, whereupon a tumor could be excised 7–21 days post injection (Figure 1A). Since the CAG promoter controls the expression of the two mutated transgenes, *KRAS^G12D^* and *TP53^R167H^*, showing the gene product of one or the other transgene is sufficient to confirm successful transformation. Therefore, the presence of *KRAS^G12D^* was shown at the protein level using intracellular flow cytometry staining of single-cell suspensions obtained from tumor biopsies (Figure 1B). Having confirmed the ability to induce tumors in the Oncopig, we then examined for the presence of intratumoral T cells. Tumor sections obtained from Oncopigs injected with AdCre at two different sites, subcutaneous (Figures 1C,D) and intramuscular (Figures 1E,F), were immunohistochemistry stained for the common T-cell marker CD3. Independent of the site of AdCre administration, CD3^+^ cells were found to infiltrate the tumors. Since the site of AdCre administration did not affect the T-cell infiltration, subcutaneous tumors were used for the remaining parts of the study.

### CD8β^+^ T Cells Preferentially Infiltrate Oncopig Tumors

**Figure 2:**
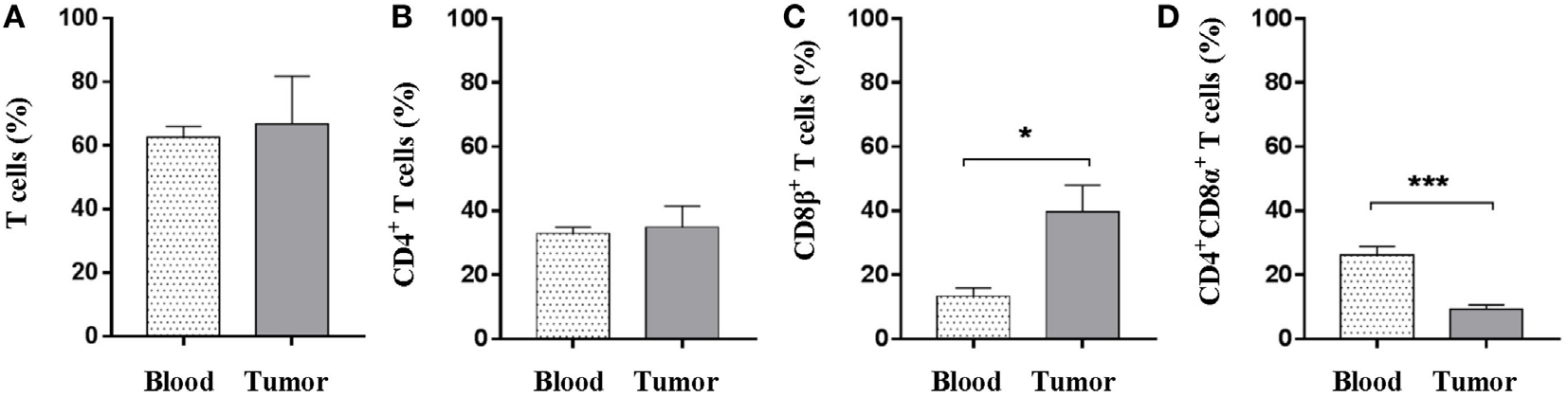
CD8β^+^ T cells specifically infiltrate the established tumors. Oncopigs were subcutaneously injected with AdCre. PBMCs and tumor tissue were harvested 7–21 days post injection and analyzed using flow cytometry. Cells were pre-gated on viable, single lymphocytes. **(A)** Numbers represent CD3^+^ cells as a percentage of live cells. **(B)** Percentage of CD4^+^ cells in live, CD3^+^-gated cells. **(C)** Percentage of CD8β^+^ cells in live, CD3^+^-gated cells. **(D)** Percentage of CD8α^+^ cells in live, CD3^+^CD4^+^-gated cells. Bars represent mean values ± SEM, and data are from two independent experiments (*n* = 4–5), where *n* indicates the number of animals. Statistical evaluations were performed by unpaired Student’s *t*-test (*P < 0.05 and ***P < 0.001).

Given that T cells infiltrate tumors as shown by immunohistochemistry, the next step was to address which T-cell subsets were present and whether the intratumoral T-cell pool differed from the circulating counterpart. Using flow cytometry, quantification of the percentage of total (CD3^+^) T cells revealed no difference between peripheral blood and tumor cell isolates (Figure 2A); thus, indicating that PBMCs and tumor cell suspensions encompass similar T cells levels. In contrast to other species, CD4^+^CD8^+^ double-positive T cells comprise a significant proportion of circulating lymphocytes in the pig (49); and the vast majority of this subset expresses the CD8α homodimer which is now associated with activation of porcine CD4^+^ T cells (50). On the other hand, the expression of the CD8α/CD8β heterodimer is linked to conventional cytolytic CD8^+^ T cells (51). Comparison of the different T-cell subsets revealed that the amount of CD4^+^ T cells, as a percentage of total CD3^+^ cells, was similar in the tumor and in peripheral blood (Figure 2B). While a significant increase in percentage of CD8β^+^ T cells was found at the tumor site (mean values: 39.7% in contrast to 13.3% for the PBMC samples) (Figure 2C), the proportion of CD4^+^ T cells expressing the CD8α^+^ activation molecule was significantly reduced within tumor isolates (Figure 2D). Combined, these data showed that Oncopig tumors were specifically infiltrated by cytotoxic CD8β^+^ T cells.

### Cytotoxic Immune Cells Are Represented in the Microenvironment of Oncopig Tumors

**Figure 3:**
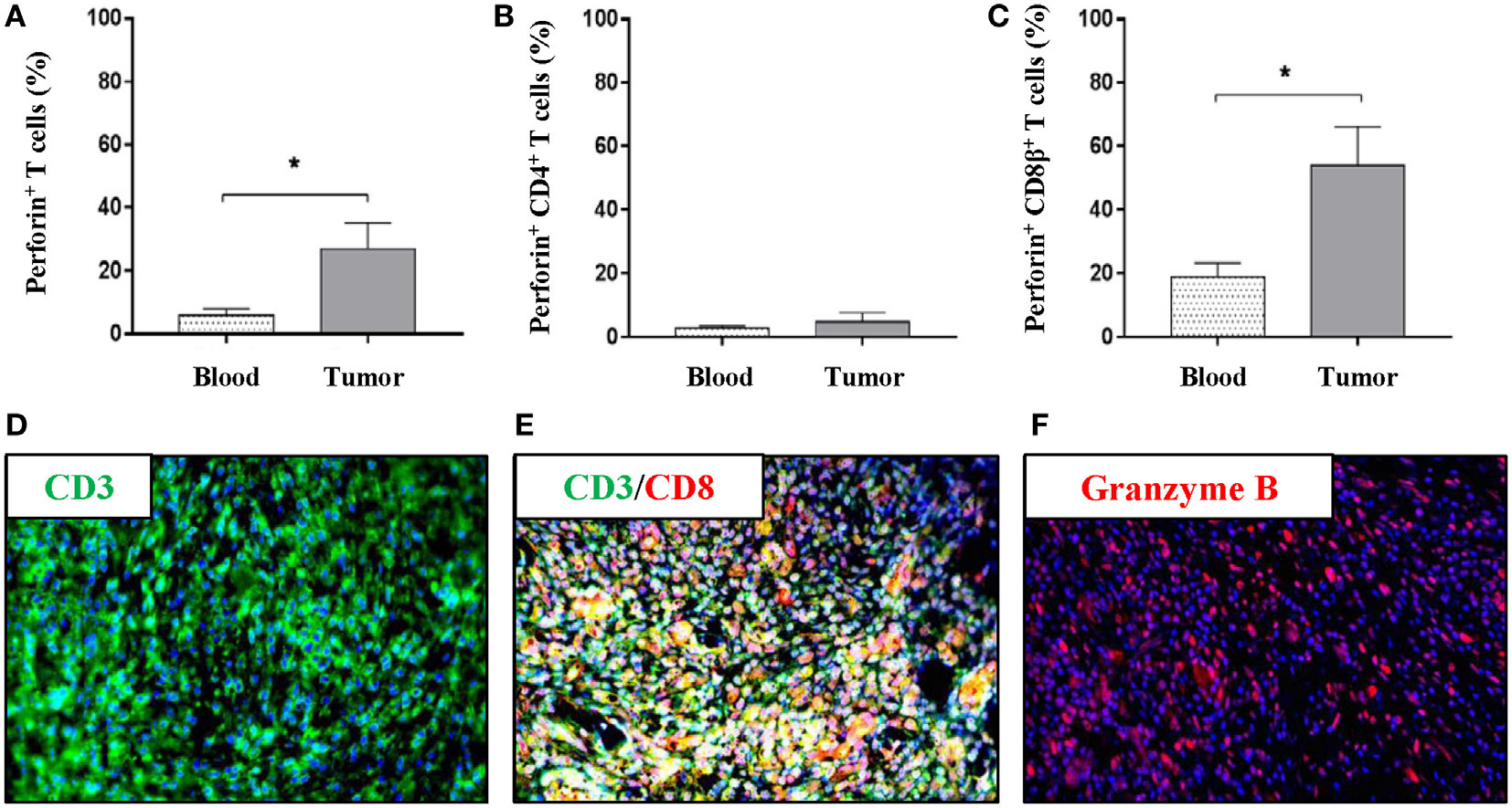
The tumor microenvironment of Oncopigs is infiltrated by perforin^+^ and granzyme B^+^ immune cells. Oncopigs were subcutaneously injected with AdCre to induce tumor formation. PBMCs and tumor samples were harvested 7–21 days post injection and analyzed by flow cytometry. Cells were pre-gated on viable, single lymphocytes. **(A)** Numbers represent perforin^+^ cells as a percentage of live CD3^+^-gated cells. **(B)** Percentage of perforin^+^ cells in live, CD3^+^CD4^+^-gated cells. **(C)** Perforin^+^ cells as a percentage of live, CD3^+^CD8β^+^-gated cells. Bars represent mean values ± SEM, and data are from two independent experiments (*n* = 4–5). Statistical evaluations were performed by unpaired Student’s *t*-test. **(D)** Tissue sections were harvested from Oncopig tumors isolated 7–21 days post AdCre injection. Detection of CD3^+^ cells (green) in a tumor cross-section by immunofluorescence. **(E)** Immunofluorescence image detecting co-localization of CD3^+^ (green) and CD8α^+^ (red) cells in the tumor. **(F)** Detection of granzyme B^+^ cells (red) in a tumor cross-section. DAPI (blue) used as nuclear counterstain for all immunofluorescence images (*P < 0.05).

To further investigate the nature of the intratumoral T-cell subsets in more detail, the production of perforin, TNF-α, and IFN-γ was evaluated in T cells derived from peripheral blood and tumor samples. Using flow cytometry, perforin-producing T cells were observed both in peripheral blood and within the tumor; however, a greater than fourfold increase in total perforin-producing T cells was observed in the tumor samples over peripheral blood samples (mean values: 26.9 vs 5.8%) (Figure 3A). T cells producing TNF-α or IFN-γ were not detectable without further stimulation (data not shown). CD4^+^ T cells, as expected, produced only very limited amounts of perforin, and no differences were observed in perforin^+^CD4^+^ T cells between the PBMC and the tumor samples (Figure 3B). By contrast, a perforin^+^CD8β^+^ T-cell population was clearly detected in the tumor with nearly a threefold increase compared with peripheral blood (Figure 3C); indicating a cytotoxic infiltration to the tumor. To further corroborate this observation, immunofluorescence using formalin-fixed tumor sections was performed. First, the infiltration of CD3^+^ cells previously observed (Figures 1C–F) was confirmed (Figure 3D). Second, co-localization of the CD3 and the CD8α marker within the tumor was demonstrated (Figure 3E). Importantly, intratumoral granzyme B^+^ cells were visualized (Figure 3F); thereby, confirming the presence of cytotoxic cells within the tumor. The percentages of CD4^+^, CD8β^+^, and perforin^+^CD8β^+^ T cells in peripheral blood did not reveal any difference between tumor bearing and non-tumor bearing pigs (Figures S2A–C in Supplementary Material). The representation of natural killer (NK) cells (CD3^−^CD4^−^CD8α^+^) revealed no significant differences between the NK-cell percentage in PBMCs and intratumoral cell isolates (Figure S2D in Supplementary Material).

### Oncopig Tumors Are Specifically Infiltrated by a Distinct Subset of γδ T Cells

**Figure 4:**
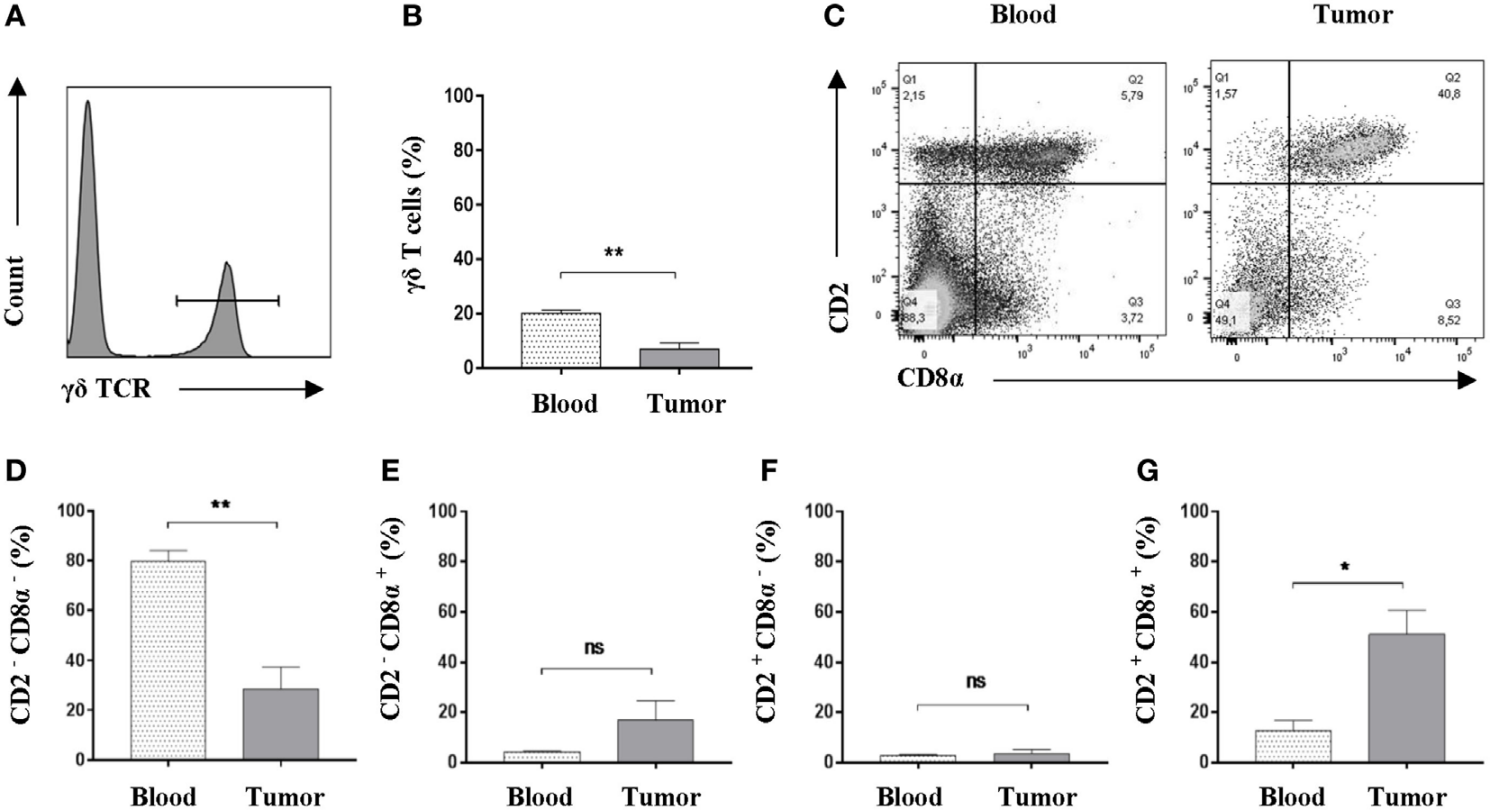
An increased representation of CD2^+^CD8α^+^ γδ T cells is found within Oncopig tumors. Oncopigs were subcutaneously injected with AdCre. Peripheral blood and tumor samples were harvested 7–21 days post injection and analyzed by flow cytometry. All cells were pre-gated on viable, single lymphocytes. **(A)** γδ T cells were detected by expression of the δ chain of the T cell receptor in viable, single lymphocytes. Representative flow cytometric plot is shown. **(B)** Representation of γδ T cells as a percentage of viable, single lymphocytes in tumor and peripheral blood. **(C)** Representative flow cytometric plots of CD2 and CD8α expression in γδ T cells obtained from peripheral blood (left plot) and tumor isolates (right). **(D)** Percentage of CD2^−^CD8α^−^ cells in viable γδ T cells. **(E)** Percentage of CD2^−^CD8α^+^ cells in viable γδ T cells. **(F)** Percentage of CD2^+^CD8α^−^ cells in viable γδ T cells. **(G)** Percentage of CD2^+^CD8α^+^ cells in viable γδ T cells. Bars represent mean ± SEM, and data are from one experiment (*n* = *5*). Statistical evaluations were performed by paired Student’s *t*-test (*P < 0.05 and **P < 0.005).

While conventional αβ T cells have received a lot of attention, γδ T cells have been much less studied, although they have been demonstrated to have implications in cancer (52). As γδ T cells represent a major porcine T-cell population (53, 54), we set to determine the potential presence of this immune cell subset in Oncopig tumors. Using flow cytometry, we once again compared peripheral blood and tumor isolates. First, and by use of an antibody detecting the δ chain of the T-cell receptor (55) (Table S1 in Supplementary Material), γδ T cells were detected in viable, single-cell suspensions (Figure 4A). A comparison between PBMC and tumor samples revealed a significant reduction in the total representation of γδ T cells within the tumor (Figure 4B). In pigs, the different γδ T-cell subsets and their functional differentiation are traditionally defined by their expression of CD2 and CD8α (56). Using flow cytometry, the expression level of CD2 and CD8α in γδ T cells was evaluated in both PBMC and tumor samples (Figure 4C). Comparison of the different γδ T-cell subsets revealed a significant decrease in the representation of CD2^−^CD8α^−^ cells in Oncopig tumors compared with blood levels (Figure 4D), while no difference between the two sites was observed when comparing CD2^−^CD8α^+^ cells (Figure 4E) or CD2^+^CD8α^−^ cells (Figure 4F). Interestingly, a fourfold increase in the percentage of γδ T cells displaying the CD2^+^CD8α^+^ phenotype was detected in tumors compared with blood (Figure 4G); mean values: 51.1% in tumor isolates in contrast to 12.8% for the PBMC samples. Combined, these data show that Oncopig tumors are infiltrated by γδ T cells with a distinct CD2^+^CD8α^+^ phenotype compared with the circulating counterpart.

### Increased Levels of FOXP3^+^ T Cells Are Found Within Oncopig Tumors

**Figure 5:**
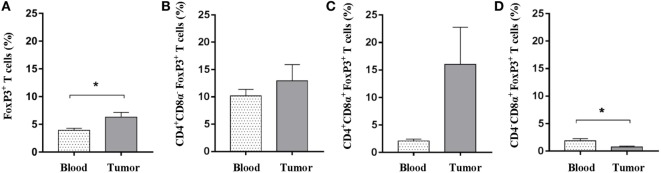
Oncopig tumors display increased levels of FOXP3^+^ T cells. Oncopigs were subcutaneously injected with AdCre. Peripheral blood and tumor samples were harvested 7–21 days post injection and analyzed for intracellular expression of FOXP3 by flow cytometry. Cells were pre-gated on viable, single lymphocytes. **(A)** Percentage of FOXP3^+^ cells in live, CD3^+^-gated cells. **(B)** Percentage of FOXP3^+^ cells in live, CD4^+^CD8α^−^ gated T cells. **(C)** Percentage of FOXP3^+^ cells in live, CD4^+^CD8α^+^-gated T cells. **(D)** Percentage of FOXP3^+^ cells in live, CD4^−^CD8α^+^-gated T cells. All bars represent mean values ± SEM, and data are from one experiment (*n* = 5). Statistical evaluations were performed by paired Student’s *t*-test (*P < 0.05).

Tumor microenvironments often contain a mixture of immune cells. In addition to the cytotoxic immune cell subsets and γδ T cells, which were shown to be present, we tested for regulatory T cells using flow cytometric detection of the FOXP3 marker. A pronounced population of T cells expressing FOXP3 was readily detected in both blood samples and within tumors. When comparing these two sites, a significant elevated representation of FOXP3^+^ T cells was detected within the tumors (Figure 5A), suggesting an intratumoral regulatory compartment. Similar percentages of CD4^+^CD8α^−^FOXP3^+^ T cells were observed when comparing blood and tumor isolates (mean values: 10.1 and 12.9%) (Figure 5B). Although not significant due to high animal to animal variation, a strong tendency toward an increased amount of CD4^+^FOXP3^+^ T cells expressing the CD8α activation marker was observed in the tumor when compared with circulating blood (mean values: 16.0 and 2.1%) (Figure 5C). By contrast, the circulating T-cell pool comprised a slightly higher amount of potential regulatory CD4^−^CD8α^+^FOXP3^+^ T cells; although the percentages were low in general (Figure 5D). Together, these data suggest that Oncopig tumors also encompass an active regulatory T-cell compartment.

### Oncopig Tumors Show Elevated *IDO1, CTLA4*, and *PDL1* Expression Levels

In addition to the presence of FOXP3^+^ T cells within Oncopig tumors, we determined if other intratumoral immunoregulatory mechanisms were present. Indoleamine 2,3-dioxygenase 1 (*IDO1)*, cytotoxic T-lymphocyte-associated protein 4 *(CTLA4*), and programmed death-ligand 1 (*PDL1)* encode for proteins that are activated during tumor development in humans and play a role in suppressing immune responses, ultimately helping malignant cells escape T-cell mediated killing. To determine if these genes are upregulated in Oncopig tumors, expression levels were investigated using previously produced Oncopig RNA-Seq datasets (28, 43, 44). As expected, increased expression of *IDO1, CTLA4*, and *PDL1* was observed in Oncopig leiomyosarcoma tumors relative to control muscle samples (Table 1). No increased expression was observed in Oncopig transformed cell lines (HCC and fibroblasts) compared with primary non-transformed cell lines, indicating the increased expression observed in Oncopig tumors is not simply a result of cellular transformation (Table S2 in Supplementary Material). Together, these data indicate a suppressive role for *IDO1, CTLA4*, and *PDL1* within Oncopig tumors.

**Table 1:** Elevated *IDO1, CTLA4*, and *PDL1* expression in Oncopig tumors.

Gene	Skeletal muscle (FPKM)	Leiomyosarcoma (FPKM)	Log2 fold change	*P*-value	*q*-Value	Significant
*IDO1*	0.49	3.8	3.0	5.00E−05	0.00023	Yes
*CTLA4*	0.13	1.0	2.9	5.00E−05	0.00023	Yes
*PDL1*	0.34	1.1	1.7	0.00075	0.0028	Yes

*Gene expression was determined by RNA-Seq analysis with comparison of normal skeletal muscle tissue and AdCre-induced leiomyosarcoma. Expression values are given as fragments per kilobase of transcript per million mapped reads (FPKM). *q*-Value <0.05 is considered significant*.

*CTLA4, cytotoxic T-lymphocyte-associated protein 4; IDO1, indoleamine 2,3-dioxygenase 1; PDL1, programmed death-ligand 1*.

### Autologous Tumor Cells Are Specifically Lysed by the Oncopig Immune System *In Vitro*

**Figure 6:**
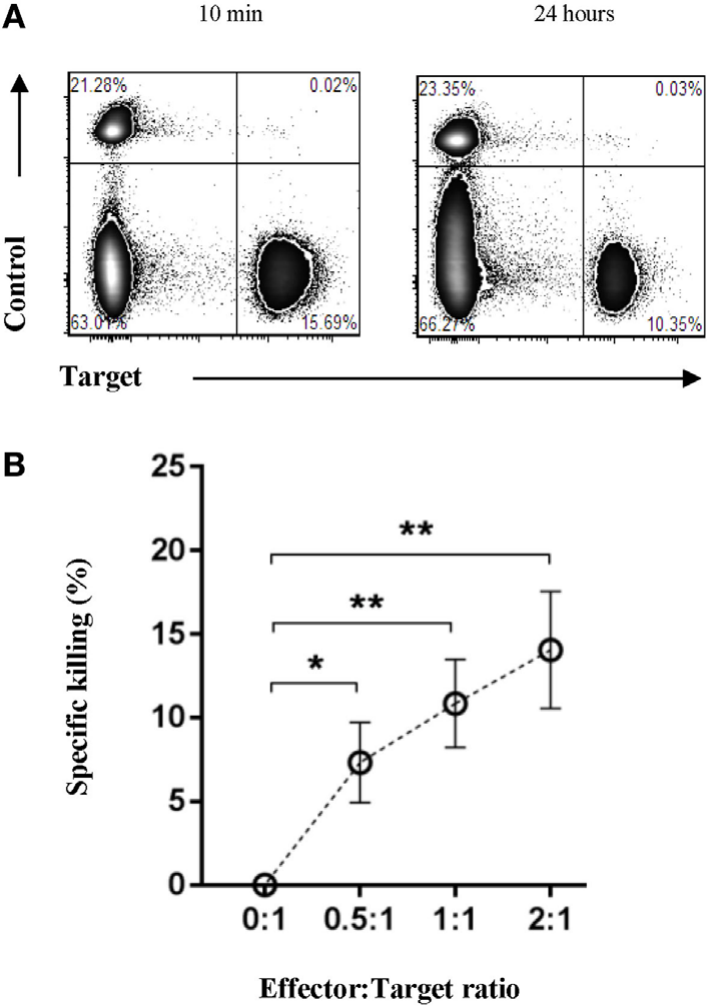
The Oncopig immune system specifically lyses autologous tumor cells *in vitro*. Oncopigs were subcutaneously injected with AdCre to induce tumor formation. Following tumor development (7–21 days post injection), tumor cells and PBMCs were harvested. Isolated effector cells remained unlabeled with control cells and tumor cells being labeled with eFluor670 or eFluor450, respectively. **(A)** Representative flow cytometric plots of control and tumor cells at 10 min (baseline, left plot) and 24 h (right plot) post coculture. **(B)** Numbers represent percentage specific killing of autologous tumor cells; data were normalized to adjust for cell turnover in no-effector cells control cultures. A titration of the effector:target cell ratio is shown. Data are pooled from four independent experiments (*n* = 8). Bars represent mean values ± SEM. Statistical evaluations were performed by paired Student’s *t*-test (*P < 0.05 and **P < 0.005).

Having shown the presence of both cytotoxic and regulatory immune cells within Oncopig tumors, we set to determine if the Oncopig immune system was capable of mediating direct antitumor immunity outside of an immunosuppressive microenvironment. For this reason, we developed an *in vitro* fluorescence-based cytotoxicity assay to allow the investigation of potential immune-mediated killing of autologous tumor cells. Isolated effector cells were cocultured with either autologous targets or autologous control cells, and specific killing was monitored by flow cytometry. PBMCs were used as control cells, since both healthy, adjacent skin and muscle cells isolated from the same site as the tumor did not allow a clear fluorescence separation. A twofold titration of the effector:target cell ratio was performed ranging from 0:1 to 2:1. To determine lysis of the tumor cells, samples were harvested 10 min (Figure 6A, left plot) and 24 h post coculture (Figure 6A, right plot), and the percentage of specific tumor cell killing was quantified relative to the 10 min baseline. Each sample was normalized to its 0:1 effector:target control sample. Significant percentages of specific tumor cell killing were observed in an effector:target cell ratio dependent manner (Figure 6B), thereby, for the first time directly showing an endogenous porcine anti-cancer immune response in the Oncopig model.

## Discussion

Although valuable, mice have several inherent limitations in cancer research. In addition to size and anatomical constraints, inbred rodents also do not fully mimic the diversity seen in human patients. Therefore, to establish a supplementary and large preclinical model, we performed our studies in the Oncopig; increasing diversity by using non-sex- and non-age-matched animals and restricting the use of littermates. Given the homology between the porcine and human immune system (24), the fully immunocompetent Oncopig may be an excellent platform studying antitumor immune responses and for preclinical investigation of cancer immunotherapies. To begin to assess the validity of the Oncopig model, we induced mutant transgene expression and tumor formation by subcutaneous delivery of AdCre. Theoretically, an increase in percentages of a certain immune cell subset within the tumor could result from either a consistent infiltration of these cells over time, intranodal proliferation, or efflux of other immune cell subsets from the tumor. For this reason, we do not conclude on exact numbers but report important differences in the representation of various T-cell subsets between the tumor and peripheral blood.

Following exposure to AdCre and tumor development, the resulting tumor microenvironment was infiltrated by T cells displaying in particular a cytotoxic phenotype as determined by the expression of CD8β, the porcine marker for cytolytic T cells (51) while activated CD4 T cells (CD4^+^CD8α^+^) were reduced relative to the representation in peripheral blood. Although antitumor immune responses are often evaluated using IFN-γ as readout, granzyme B and perforin release are two highly specific measures of antitumor cytotoxicity (57–61). We observed pronounced intratumoral granzyme B production and increased levels of perforin-producing T cells within Oncopig tumors. Finally, we showed the capacity of the Oncopig immune system to mediate tumor-specific lysis *in vitro*; further supporting the presence of an adaptive antitumor immune response. Although we show pronounced infiltration of various T-cell subsets to the tumors, the antitumor immune responses demonstrated in our *in vitro* cytotoxicity could be mediated by other immune cell subsets present in the PBMC culture. Potential other subsets, which might mediate the antitumor response, include NK cells, γδ T cells, and natural killer T cells. In fact, porcine NK cells have been shown to display antitumor activities against a human cancer cell line (62); however, we did not observe *in vivo* specific NK-cell infiltration to the tumor site compared with the representation found in circulation. Despite not specifically enriched in the tumor, these intratumoral NK cells may still play a role. As T cells are key players in mediating antitumor immune responses (63–65), the significant infiltration of cytotoxic T cells and highly differentiated γδ T cells to Oncopig tumors clearly suggest a role for these immune cell subsets in facilitating tumor-specific lysis. To fully evaluate the role of conventional T cells in Oncopig antitumor immunity, blocking the MHC presentation pathway will be an interesting future approach as well as depletion studies of the various T-cell subsets.

In addition to the infiltration of cytotoxic T cells, we showed intratumoral enrichment of a CD2^+^CD8α^+^ γδ T-cell subset. Traditionally, porcine γδ T cells have been divided into three distinct subsets based on their expression of the CD2 and CD8α markers, including CD2^−^CD8α^−^, CD2^+^CD8α^−^, and CD2^+^CD8α^+^ cells (56). The CD2^−^CD8α^+^ cells remain undescribed. Our data support previous findings showing that the CD2^−^CD8α^−^ cells are the most commonly found γδ T cells in circulating porcine blood (66, 67). This subset has been reported to comprise up to 90% of the total γδ T-cell population in peripheral blood (68), which is in line with our results (mean value: 79.9%). By contrast, CD2^+^CD8α^−^ and CD2^+^CD8α^+^ γδ T cells have been reported to preferentially home to the lymphoid tissues (66). As the CD2^+^CD8α^−^ cells become more differentiated, they acquire CD8α expression (69); consequently, the CD2^+^CD8α^+^ cells are considered the highly differentiated γδ T-cell subset (56, 68). Although γδ T cells have been shown to have implications in cancer, the nature of the tumor antigens recognized by γδ T cells remains fairly unknown in both human cancer and murine tumor models (52). The significant enrichment of highly differentiated γδ T cells found in Oncopig tumors clearly supports a role for this immune cell subset, and future studies should elucidate their specific role within tumor microenvironment.

FOXP3^+^ T cells are common regulators of cytotoxic T-cell responses, and high levels of peripheral CD4^+^CD25^+^FOXP3^+^ regulatory T cells have been associated with poor clinical response to adoptive cell therapy in human cancer (70). We observed a robust subpopulation of T cells expressing FOXP3, both systemically as well as in the induced tumors. Recent findings suggest that human T helper cells can transiently upregulate FOXP3 upon activation, although only the T cells stably expressing FOXP3 were found to exhibit a suppressive nature (71). Therefore, the detection of FOXP3 in various intratumoral T-cell subsets in the Oncopig might indicate the presence of newly activated T cells. However, it is well established that FOXP3 is required for the development and maintenance of suppressive regulatory T cells (72, 73). Moreover, FOXP3 has been suggested as an exclusive marker for the CD4^+^CD25^+^ regulatory T-cell lineage in mice (74), and a suppressive CD8α^+^CD25^+^FOXP3^+^ T-cell subset has recently been observed in both mice and humans (75). The significant infiltration of FOXP3-expressing T cells to the tumor mass suggest a regulatory role for this these immune cells in Oncopig tumors. In addition to the FOXP3^+^ T cells, increased expression of the immunosuppressive genes *IDO1, CTLA4*, and *PDL1* was observed in Oncopig tumors but not in Oncopig-derived cell lines transformed *in vitro*. The lack of elevated expression *in vitro* indicates these genes are not simply upregulated as a result of cellular transformation, but rather in response to signals from the *in vivo* tumor microenvironment. The increased expression of *IDO1, CTLA4*, and *PDL1* in Oncopig tumors thus indicates suppression of T cells *in vivo*. Another important immune cell subset with the capacity to dampen antitumor T-cell responses is the myeloid-derived suppressor cells (MDSCs). Within the tumor microenvironment, MDSCs are often present and efficiently inhibit effector T-cell function by depletion of cysteine; an essential amino acid for T-cell activation (76). Moreover, MDSCs can limit T-cell functions by production of reactive oxygen species, arginase, and nitric oxide (77–79). Future studies should evaluate the role of these innate immune cells within Oncopig tumors.

Altogether, these findings suggest that within Oncopig tumors an antitumor immune response, dominated by cytotoxic T cells and differentiated γδ T cells, develop in parallel with a regulatory response mediated by FOXP3^+^ T cells and elevated expression of immunosuppressive genes. As a spontaneous porcine model of melanoma displays a high rate of tumor regression over time (19), it will be important to investigate whether this antitumor immunity shown here *in vitro* becomes dominant over time in the Oncopig model or remains suppressed *in vivo*.

The porcine immunome and inflammasome shares a large homology with humans while the murine set of immune response genes is characterized by redundancy in terms of addition of many unique genes (24, 80). Other human to pig differences include the larger proportion of circulating γδ T cells and CD4^+^CD8^+^ T cells in porcine peripheral blood (50), and anatomical differences such as the inversion of the lymph node architecture, with resulting difference in lymphocyte recirculation, and a distinct porcine ileal Peyer’s patch (81). Importantly, it remains to be determined how these similarities and differences in gene expression circulating lymphocyte subsets and anatomy are reflected biological function.

In conclusion, we for the first time showed that the Oncopig immune system is capable of recognizing the AdCre-induced tumors and responding with the development of antitumor responses specifically able to lyse autologous tumor cells *in vitro* as well as immunological regulatory responses in line with known escape mechanisms for cancer immunoediting (82). Combined, we believe that the Oncopig with its fully competent immune system and homology with humans provides a crucial platform for studying antitumor immune responses with potential for future preclinical testing of immunotherapies aimed at reactivating the antitumor immune responses observed *in vitro*.

## Data Availability Statement

The raw data supporting the conclusions of this manuscript will be made available by the authors, without undue reservation, to any qualified researcher.

## Ethics Statement

All animal experiments were carried out in accordance with both national and international guidelines. The University of Illinois Institutional Animal Care and Use Committee (IACUC; Protocol number 14126) approved all procedures.

## Author Contributions

Conceived and designed the experiments: NO, LR, LS, and GJ. Performed the experiments: NO, DP, JJ, and LR. Data analysis and interpretation: NO, DP, KS, JJ, LR, LS, and GJ. Manuscript preparation: NO, DP, KS, JJ, LR, PG, LS, and GJ.

## Conflict of Interest Statement

The authors declare that the research was conducted in the absence of any commercial or financial relationships that could be construed as a potential conflict of interest.

## Footnotes

^1^www.ebi.ac.uk/ena (Accessed: September 12, 2017).

^2^www.ebi.ac.uk/arrayexpress (Accessed: September 12, 2017).

^3^http://www.bioinformatics.babraham.ac.uk/projects/trim_galore/ (Accessed: September 12, 2017).

## Abbreviations

AdCre, adenoviral vector cre-recombinase; CTLA4, cytotoxic T-lymphocyte-associated protein 4; DMSO, dimethyl sulfoxide; FBS, fetal bovine serum; IDO1, indoleamine 2,3-dioxygenase 1; MDSCs, myeloid-derived suppressor cells; NK, natural killer; PMBCs, peripheral blood mononuclear cells; PBS, phosphate buffered saline; PDL1, programmed death-ligand 1.
