# Supplementary material for: Genetically Induced Tumors in the Oncopig Model Invoke an Antitumor Immune Response Dominated by Cytotoxic CD8β+ T Cells and Differentiated γδ T Cells Alongside a Regulatory Response Mediated by FOXP3+ T Cells and Immunoregulatory Molecules
Source: Front Immunol. 2018 Jun 7;9:1301. doi: 10.3389/fimmu.2018.01301 (PMC5999797; doi:10.3389/fimmu.2018.01301)
Supplement: Supplementary file 1 [file image_1.PDF]

Supplementary Figure 1

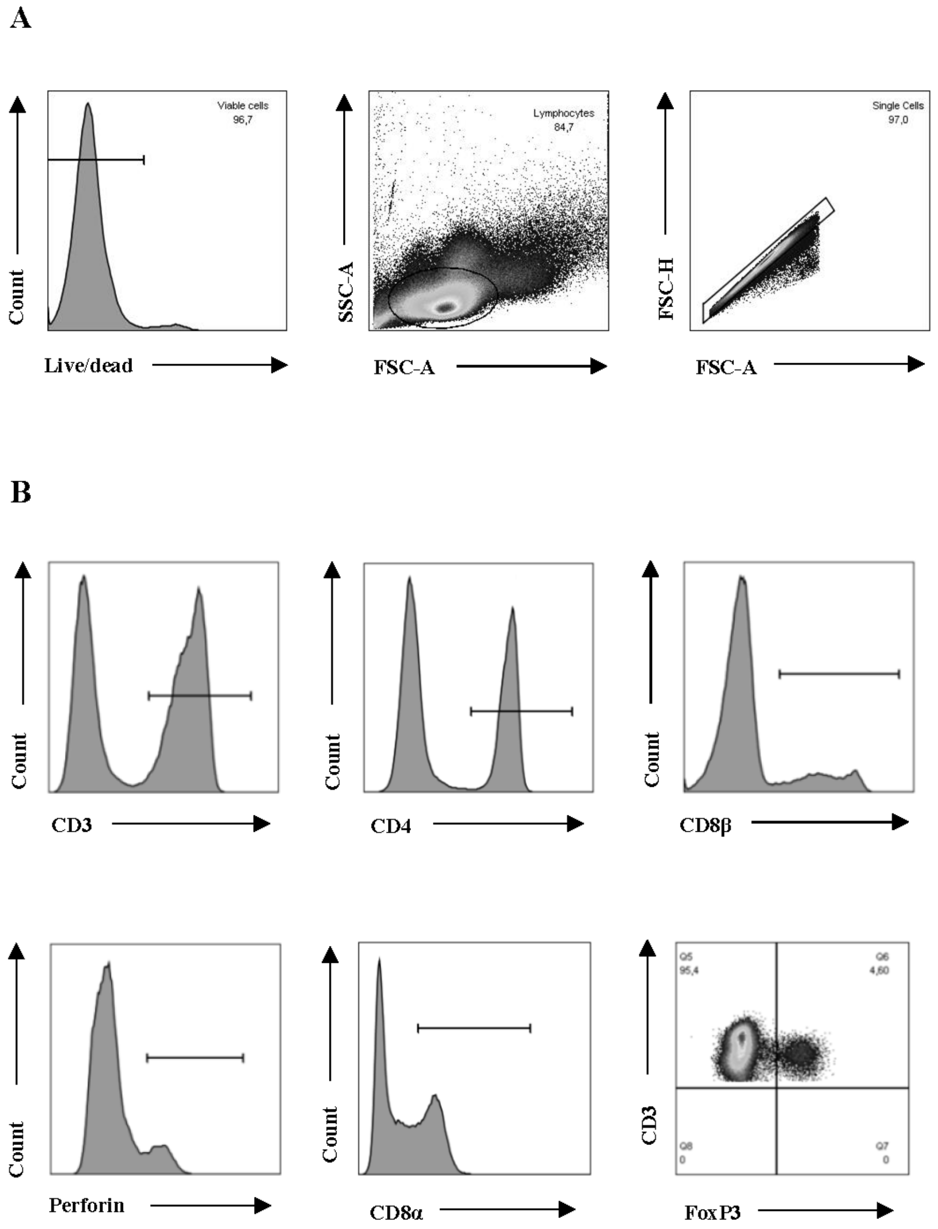

Supplementary Figure 1. Gating strategy and representative flow cytometric plots. (A) Representative gating strategy used for flow cytometry. All cells were pre-gated on viable cells following selection of single lymphocytes. (B) Representative flow cytometric plots showing staining of $CD3^+$, $CD4^+$, $CD8\beta^+$, $Perforin^+$, $CD8\alpha^+$, or $FoxP3^+$ T cells. Data is shown using a representative PBMC sample. The same approach was used for all tumor samples.
