# Supplementary material for: Genetically Induced Tumors in the Oncopig Model Invoke an Antitumor Immune Response Dominated by Cytotoxic CD8β+ T Cells and Differentiated γδ T Cells Alongside a Regulatory Response Mediated by FOXP3+ T Cells and Immunoregulatory Molecules
Source: Front Immunol. 2018 Jun 7;9:1301. doi: 10.3389/fimmu.2018.01301 (PMC5999797; doi:10.3389/fimmu.2018.01301)
Supplement: Supplementary file 2 [file image_2.PDF]

Supplementary Figure 2

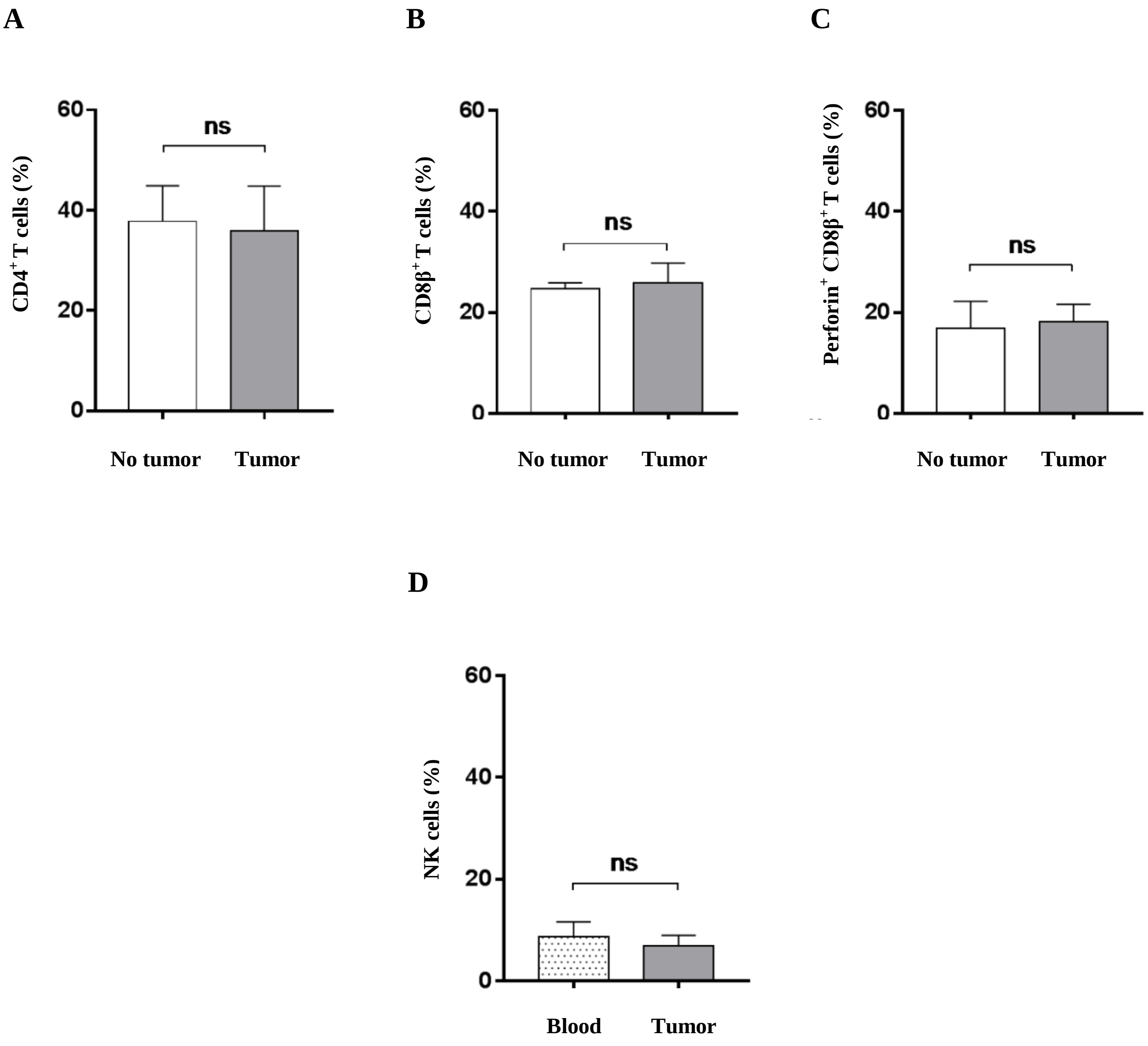

Supplementary Figure 2. Comparison of the systemic T-cell compartment in tumor-bearing and non-tumor-bearing animals as well as detection of natural killer cells in Oncopig tumors. Peripheral blood samples from tumor-bearing and healthy controls (non-tumor-bearing) were harvested for comparison of their T-cell compartments. (**A**) $CD4^+$ T cells as a percentage of total live, $CD3^+$ cells. (**B**) Percentage of $CD8\beta^+$ T cells as a proportion of total live, $CD3^+$ cells. (**C**) Percentage of $perforin^+$ cells as a proportion of live, single $CD3^+CD8\beta^+$ cells. Bars represent mean ± SEM and data are from one experiment (*n=3*). Statistical evaluation in (A), (B), and (C) was performed by unpaired Student's t-test. (**D**) Peripheral blood samples and tumor cell isolates were harvested for flow cytometric detection of natural killer (NK) cells. Numbers represent $CD3^-CD4^-CD8\alpha^+$ cells as a proportion of live, single cells. Bars represent mean ± SEM and data are from one experiment (*n=3*). Statistical evaluation in (D) was performed by paired Student's t-test.
