# Supplementary material for: Genetically Induced Tumors in the Oncopig Model Invoke an Antitumor Immune Response Dominated by Cytotoxic CD8β+ T Cells and Differentiated γδ T Cells Alongside a Regulatory Response Mediated by FOXP3+ T Cells and Immunoregulatory Molecules
Source: Front Immunol. 2018 Jun 7;9:1301. doi: 10.3389/fimmu.2018.01301 (PMC5999797; doi:10.3389/fimmu.2018.01301)
Supplement: Supplementary file 3 [file table_1.PDF]

Supplementary Table 1. Antibodies used for flow cytometry. The antibodies were used at predetermined optimal concentrations (dilution factor shown).

Marker	Conjugate	Isotype	Clone	Dilution	Supplier	Catalogue number
CD2	Biotin	IgG2a	MSA-4	1 : 100	ATCC	PG2007 cell line
CD3	Unconjugated	Mouse IgG1	PPT3	1 : 100	Southern Biotech	4510-01
CD3	FITC	Mouse IgG1	PPT3	1 : 100	Southern Biotech	4510-02
CD4	FITC	Mouse IgG2b	74-12-4	1 : 100	BD Biosciences	559585
CD4	PE-Cy7	Mouse IgG2b	74-12-4	1 : 100	BD Biosciences	561473
CD4	PerCP-Cy5.5	Mouse IgG2b	74-12-4	1 : 100	BD Biosciences	561474
CD8 α	AF647	Mouse IgG2aκ	76-2-11	1 : 100	BD Biosciences	561475
CD8 α	PE	Mouse IgG2aκ	76-2-11	1 : 100	BD Biosciences	559584
CD8 β	Unconjugated	Mouse IgG2a	PG164A	1 : 100	Washington State University	PG2020
Live/Dead	Aqua	N/A	N/A	1 : 1000	Thermo Fischer Scientific	L34957
IFN- γ	AF647	Mouse IgG1	CC302	1 : 20	Serotec	MCA1783A647
TNF- α	PerCP-Cy5.5	Mouse IgG1κ	MAb11	1 : 20	Biolegend	502926
Perforin	PE	Mouse IgG2bκ	dG9	1 : 20	Biolegend	308106
FoxP3	PE	Rat IgG2aκ	FJK-16s	1 : 50	eBioscience	12-5773-82
TCR1 δ chain	Unconjugated	IgG1	PGBL22A	1 : 100	Washington State University	WS0621S-100
IgG2a goat anti-mouse	PE-Cy7	Goat IgG	N/A	1 : 200	Southern Biotech	1080-17
IgG1 rat anti-mouse	BV421	Rat LOU	N/A	1 : 500	BD Biosciences	562580
Streptavidin	PE	N/A	N/A	1 : 200	BD Biosciences	554061
RASG12D	Unconjugated	IgG2a	N/A	1 : 100	NewEast Biosciences	26036
