# Supplementary material for: Genetically Induced Tumors in the Oncopig Model Invoke an Antitumor Immune Response Dominated by Cytotoxic CD8β+ T Cells and Differentiated γδ T Cells Alongside a Regulatory Response Mediated by FOXP3+ T Cells and Immunoregulatory Molecules
Source: Front Immunol. 2018 Jun 7;9:1301. doi: 10.3389/fimmu.2018.01301 (PMC5999797; doi:10.3389/fimmu.2018.01301)
Supplement: Supplementary file 4 [file table_2.PDF]

Supplementary Table 2. *IDO1*, *CTLA4*, and *PDL1* expression in Oncopig cell lines. Expression values are given as fragments per kilo base of transcript per million mapped reads (FPKM). q-value < 0.05 is considered significant.

Gene	Primary Hepatocytes (FPKM)	HCC Cell Lines (FPKM)	Log2 fold change	P-value	Q-value	Significant
IDO1	1.2	0.041	-4.8	0.15	0.23	no
CTLA4	0	0	0	1	1	no
PDL1	1.2	1.5	0.41	0.28	0.37	no
**Gene**	**Primary Fibroblasts (FPKM)**	**Transformed Fibroblasts (FPKM)**	**Log2 fold change**	**P-value**	**Q-value**	**Significant**
IDO1	0.017	0.68	5.3	0.32	0.53	no
CTLA4	0	0	0	1	1	no
PDL1	0.46	0.31	-0.57	0.39	0.60	no

Abbreviations: CTLA4, Cytotoxic T-lymphocyte-associated protein 4; HCC, Hepatocellular carcinoma; IDO1, Indoleamine 2,3-dioxygenase 1; PDL1, Programmed death-ligand 1.
