# Supplementary material for: Genetically Induced Tumors in the Oncopig Model Invoke an Antitumor Immune Response Dominated by Cytotoxic CD8β+ T Cells and Differentiated γδ T Cells Alongside a Regulatory Response Mediated by FOXP3+ T Cells and Immunoregulatory Molecules
Source: Front Immunol. 2018 Jun 7;9:1301. doi: 10.3389/fimmu.2018.01301 (PMC5999797; doi:10.3389/fimmu.2018.01301)
Supplement: Supplementary file 5 [file table_3.PDF]

Supplementary Table 3. RNA sequencing depth.

Sample	# Reads
**Hepatocyte Cell Lines**	
Sample 1	28,385,664
Sample 2	30,286,514
Sample 3	23,391,879
**HCC Cell Lines**	
Sample 1	31,047,086
Sample 2	32,296,259
Sample 3	29,239,571
**Fibroblast Cell Lines**	
Sample 1	29,929,073
Sample 2	35,033,512
Sample 3	32,329,908
Sample 4	39,461,197
Sample 5	38,850,155
Sample 6	39,875,177
Sample 7	38,436,787
Sample 8	33,878,025
**Soft-tissue Sarcoma Cell Lines**	
Sample 1	31,158,267
Sample 2	33,570,179
Sample 3	32,627,915
Sample 4	31,471,232
**Skeletal Muscle**	
Sample 1	54,636,264
Sample 2	69,048,606
Sample 3	64,321,325
**Leiomyosarcoma Tumors**	
Sample 1	84,736,168
Sample 2	84,016,260
Sample 3	66,038,422
Sample 4	67,842,129
